# Supplementary material for: Etiology of gender incongruence and its levels of evidence: A scoping review protocol
Source: PLoS One. 2023 Mar 13;18(3):e0283011. doi: 10.1371/journal.pone.0283011 (PMC10010510; doi:10.1371/journal.pone.0283011)
Supplement: S1 Appendix — (DOCX) [file pone.0283011.s001.docx]

Data extraction instrument

| Author/s |  |
| --- | --- |
| Year of publication |  |
| Title |  |
| Journal |  |
| Volume |  |
| Issue |  |
| Pages |  |
| Sample’s country |  |
| Context |  |
| Methodological design |  |
| Summary of the source contribution for the understanding of GI’s etiology |  |
| Biological factors suggested in the manuscript (if any) |  |
| Social/cultural factors suggested in the manuscript (if any) |  |
| Psychological/internal factors suggested in the manuscript (if any) |  |
| Other factors suggested in the manuscript (if any) |  |
| Level of evidence of each factor suggested in the manuscript |  |
