# Supplementary material for: Etiology of gender incongruence and its levels of evidence: A scoping review protocol
Source: PLoS One. 2023 Mar 13;18(3):e0283011. doi: 10.1371/journal.pone.0283011 (PMC10010510; doi:10.1371/journal.pone.0283011)
Supplement: S2 Appendix — (DOCX) [file pone.0283011.s002.docx]

Initial literature search (PubMed)

(“etiology” OR “aetiology”) AND (“sex reassignment” OR “gender reassignment" OR ”gender identity disorder" OR “sexual identity disorder” OR "gender dysphoria" OR "gender incongruence" OR "gender identity" OR “sexual identity” OR "transgender*" OR "transsexual*")
