# Supplementary material for: Etiology of gender incongruence and its levels of evidence: A scoping review protocol
Source: PLoS One. 2023 Mar 13;18(3):e0283011. doi: 10.1371/journal.pone.0283011 (PMC10010510; doi:10.1371/journal.pone.0283011)
Supplement: Attachment — Submitted filename: Response to Reviewers.docx [file pone.0283011.s004.docx]

**Response to reviewers**

**This document contains the publisher's review, peer review and our response to each of the suggestions (in bold).**

PONE-D-22-26911

Etiology of Gender Incongruence and its levels of evidence: a scoping review protocol

PLOS ONE

Dear Dr. Rojas Saffie,

Thank you for
Please submit your revised manuscript by Jan 28 2023 11:59PM. If you will need more time than this to complete your revisions, please reply to this message or contact the journal office at [plosone@plos.org](mailto:plosone@plos.org). When you're ready to submit your revision, log on to <https://www.editorialmanager.com/pone/> and select the 'Submissions Needing Revision' folder to locate your manuscript file.

Please include the following items when submitting your revised manuscript:

- A rebuttal letter that responds to each point raised by the academic editor and reviewer(s). You should upload this letter as a separate file labeled 'Response to Reviewers'.
- A marked-up copy of your manuscript that highlights changes made to the original version. You should upload this as a separate file labeled 'Revised Manuscript with Track Changes'.
- An unmarked version of your revised paper without tracked changes. You should upload this as a separate file labeled 'Manuscript'.

If you would like to make changes to your financial disclosure, please include your updated statement in your cover letter. Guidelines for resubmitting your figure files are available below the reviewer comments at the end of this letter.

If applicable, we recommend that you deposit your laboratory protocols in [protocols.io](http://protocols.io/) to enhance the reproducibility of your results. Protocols.io assigns your protocol its own identifier (DOI) so that it can be cited independently in the future. For instructions see: <https://journals.plos.org/plosone/s/submission-guidelines#loc-laboratory-protocols>. Additionally, PLOS ONE offers an option for publishing peer-reviewed Lab Protocol articles, which describe protocols hosted on [protocols.io](http://protocols.io/). Read more information on sharing protocols at <https://plos.org/protocols?utm_medium=editorial-email&utm_source=authorletters&utm_campaign=protocols>.

We look forward to receiving your revised manuscript.

Kind regards,

Johannes Hönekopp
Academic Editor
PLOS ONE

**ANSWER: Following the three highly appreciated revisions, we modified our MS. We hope that this new version complies with the journal standards.**

Journal Requirements:
When submitting your revision, we need you to address these additional requirements.

1. Please ensure that your manuscript meets PLOS ONE's style requirements, including those for file naming. The PLOS ONE style templates can be found at

<https://journals.plos.org/plosone/s/file?id=wjVg/PLOSOne_formatting_sample_main_body.pdf>  and

<https://journals.plos.org/plosone/s/file?id=ba62/PLOSOne_formatting_sample_title_authors_affiliations.pdf>

**ANSWER: We have checked the templates and made the adjustments to meet the journal requirements.**

2. We note that you have stated that you will provide repository information for your data at acceptance. Should your manuscript be accepted for publication, we will hold it until you provide the relevant accession numbers or DOIs necessary to access your data. If you wish to make changes to your Data Availability statement, please describe these changes in your cover letter and we will update your Data Availability statement to reflect the information you provide.

**ANSWER: On this point I sent an email requesting clarification, considering that our manuscript consists of a literature review and that, therefore, the data are available on the internet. They replied that, after reviewing it, they realized that our Data Availavility Statement was OK: “1. Your current Data Availability Statement is perfectly fine—I'm sorry for the confusion! The staff member who checked your statement unfortunately overlooked the fact that your article is a Registered Report Protocol for a systematic review, and therefore would not be providing data at this point in the conventional sense.”**

3. Please include captions for your Supporting Information files at the end of your manuscript, and update any in-text citations to match accordingly. Please see our Supporting Information guidelines for more information: <http://journals.plos.org/plosone/s/supporting-information>.

**ANSWER: We have checked the guidelines and made the adjustments to meet the journal requirements. In addition, we made a change in the numerical order of the appendices, to adapt them in order of appearance in the new manuscript. Finally, we made a change in the appendix related to the "Systematic Review and Meta-analysis: Extension for Scoping Review (PRISMA-ScR)", in which we had inadvertently introduced two checklists, when only one of them was necessary.**

4. Please upload a new copy of Figure 1 as the detail is not clear. Please follow the link for more information: <https://blogs.plos.org/plos/2019/06/looking-good-tips-for-creating-your-plos-figures-graphics/>" <https://blogs.plos.org/plos/2019/06/looking-good-tips-for-creating-your-plos-figures-graphics/>

**ANSWER: We have checked the links and made the adjustments to meet the journal requirements.**

5. Please review your reference list to ensure that it is complete and correct. If you have cited papers that have been retracted, please include the rationale for doing so in the manuscript text, or remove these references and replace them with relevant current references. Any changes to the reference list should be mentioned in the rebuttal letter that accompanies your revised manuscript. If you need to cite a retracted article, indicate the article’s retracted status in the References list and also include a citation and full reference for the retraction notice.

**ANSWER: We have checked the reference list. We have not found any retracted items. However, due to reviewers' suggestions, we have added some new articles to the reference list and withdrawn a couple:**

**This list of articles was added:**

- Palveluvalikoimaneuvoston suositus. Alaikäisten sukupuoli-identiteetin variaatioihin liittyvän dysforian lääketieteelliset hoitomenetelmät [Recommendation of the Council for Choices in Health Care in Finland (PALKO/COHERE Finland) Medical Treatment Methods for Dysphoria Related to Gender Variance In Minors] [Internet]. Helsinki: PALKO/COHERE); 2020 [cited 2023 Jan 9] p. 14. Report No.: STM038. Available from: [https://palveluvalikoima.fi/documents/1237350/22895008/Alaikäiset_suositus.pdf/c987a74c-dfac-d82f-2142-684f8ddead64/Alaikäiset_suositus.pdf?t=1592317701000](https://palveluvalikoima.fi/documents/1237350/22895008/Alaik%C3%A4iset_suositus.pdf/c987a74c-dfac-d82f-2142-684f8ddead64/Alaik%C3%A4iset_suositus.pdf?t=1592317701000)
- Care of children and adolescents with gender dysphoria – Summary [Internet]. Sweden: Socialstyrelsen; 2022 Mar [cited 2023 Jan 3]. Available from: <https://www.socialstyrelsen.se/globalassets/sharepoint-dokument/artikelkatalog/kunskapsstod/2022-3-7799.pdf>
- Cass H. Response to NHS England consultation: Draft interim service specification for specialist gender dysphoria services for children and young people [Internet]. 2022 [cited 2023 Jan 9]. Available from: <https://cass.independent-review.uk/publications/>
- Cass H. The Cass Review - Independent review of gender identity services for children and young people: Interim Report [Internet]. England; 2022 Feb [cited 2023 Jan 9]. Available from: <https://cass.independent-review.uk/publications/interim-report/>
- Cass H. Entry 8 – Beyond the Headlines – Cass Review [Internet]. [cited 2023 Jan 9]. Available from: <https://cass.independent-review.uk/entry-8-beyond-the-headlines/>
- de Vries ALC. Challenges in Timing Puberty Suppression for Gender-Nonconforming Adolescents. Pediatrics. 2020 Oct 1;146(4):e2020010611.
- Arnoldussen M, de Rooy FBB, de Vries ALC, van der Miesen AIR, Popma A, Steensma TD. Demographics and gender-related measures in younger and older adolescents presenting to a gender service. Eur Child Adolesc Psychiatry [Internet]. 2022 Nov 12 [cited 2023 Jan 9]; Available from: <https://link.springer.com/10.1007/s00787-022-02082-8>
- Abbruzzese E, Levine SB, Mason JW. The Myth of “Reliable Research” in Pediatric Gender Medicine: A critical evaluation of the Dutch Studies—and research that has followed. Journal of Sex & Marital Therapy. 2023 Jan 2;1–27.
- Pazos Guerra M, Gómez Balaguer M, Gomes Porras M, Hurtado Murillo F, Solá Izquierdo E, Morillas Ariño C. Transsexuality: Transitions, detransitions, and regrets in Spain. Endocrinol Diabetes Nutr (Engl Ed). 2020 Nov;67(9):562–7.

**This list of articles was withdrawn:**

- Cass H. Interim report [Internet]. Cass Review. The Cass review; 2022 [cited 2022Sep26]. Available from: <https://cass.independent-review.uk/publications/interim-report/>
- Vandenbussche E. Detransition-Related Needs and Support: A Cross-Sectional Online Survey. Journal of Homosexuality. 2021;69(9):1602-1620.
- Irwig M. Detransition Among Transgender and Gender-Diverse People—An Increasing and Increasingly Complex Phenomenon. The Journal of Clinical Endocrinology & Metabolism. 2022.

Reviewers' comments:

Reviewer's Responses to Questions

Comments to the Author

1. Does the manuscript provide a valid rationale for the proposed study, with clearly identified and justified research questions?

The research question outlined is expected to address a valid academic problem or topic and contribute to the base of knowledge in the field.

Reviewer #1: Yes

Reviewer #2: Yes

2. Is the protocol technically sound and planned in a manner that will lead to a meaningful outcome and allow testing the stated hypotheses?

The manuscript should describe the methods in sufficient detail to prevent undisclosed flexibility in the experimental procedure or analysis pipeline, including sufficient outcome-neutral conditions (e.g. necessary controls, absence of floor or ceiling effects) to test the proposed hypotheses and a statistical power analysis where applicable. As there may be aspects of the methodology and analysis which can only be refined once the work is undertaken, authors should outline potential assumptions and explicitly describe what aspects of the proposed analyses, if any, are exploratory.

Reviewer #1: Partly

Reviewer #2: Partly

**ANSWER: We have been grateful for all the reviewers' methodological suggestions, and in almost all cases, we have modified our MS accordingly. We have the impression that some of the reviewers' concerns stem from a certain lack of knowledge of the "Scoping Review" methodology, which has very specific and standardized guidelines (https://jbi.global/scoping-review-network/resources). On some occasions the reviewers proposed the incorporation of information that according to these guidelines is not necessary. Despite this situation, we have still chosen to incorporate some of these proposals, with the objective of presenting an MS that is even more complete than is strictly necessary. We hope that our response to the reviewers will be perceived as clear and will be well received.**

3. Is the methodology feasible and described in sufficient detail to allow the work to be replicable?

Reviewer #1: Yes

Reviewer #2: Yes

4. Have the authors described where all data underlying the findings will be made available when the study is complete?

The PLOS Data policy requires authors to make all data underlying the findings described in their manuscript fully available without restriction, with rare exception, at the time of publication. The data should be provided as part of the manuscript or its supporting information, or deposited to a public repository. For example, in addition to summary statistics, the data points behind means, medians and variance measures should be available. If there are restrictions on publicly sharing data—e.g. participant privacy or use of data from a third party—those must be specified.

Reviewer #1: Yes

Reviewer #2: No

**ANSWER: We do not know the reasons why reviewer #2 responded that we have not described where the data behind the findings will be available. It is possible that his opinion did not consider that our manuscript is based on published articles, since it is a literature review, and not on clinical data. Something similar happened when the guest editor pointed out that our Data Availability Statement was not clear: when we explained by email that our manuscript was a literature review and not a study based on clinical data, he replied that it was all in order, because our manuscript “would not be providing data at this point in the conventional sense”. Considering that our research is based on published articles, it can be stated that our data is public and available to anyone.**

5. Is the manuscript presented in an intelligible fashion and written in standard English?

PLOS ONE does not copyedit accepted manuscripts, so the language in submitted articles must be clear, correct, and unambiguous. Any typographical or grammatical errors should be corrected at revision, so please note any specific errors here.

Reviewer #1: Yes

Reviewer #2: Yes

6. Review Comments to the Author

Please use the space provided to explain your answers to the questions above and, if applicable, provide comments about issues authors must address before this protocol can be accepted for publication. You may also include additional comments for the author, including concerns about research or publication ethics.

You may also provide optional suggestions and comments to authors that they might find helpful in planning their study.

(Please upload your review as an attachment if it exceeds 20,000 characters)

Reviewer #1: This is a well-written and clearly justified protocol for a forthcoming systematic review. The research question has a good rationale which is well explained. The methods could be developed a little further, as well as the planned reporting of results. Overall I am looking forward to seeing the final review published. I have some specific points below.

**Answer: We appreciate the good overall appreciation of reviewer #1. Regarding the methodology and results, we have tried to develop them as she suggested. Changes to the manuscript will be detailed in the responses to the more specific suggestions below.**

Abstract

- reconsider use of term ‘narrative review’ as a systematic review can include narrative synthesis. The use of a narrative approach does not in itself obviate systematicity.

**Answer: We appreciate this suggestion. To remedy the assumption that all narrative reviews lack systematicity, we have added the adjective "non-systematic" to the expression "narrative review". In this way, we specify that we are referring only to narrative reviews that lack systematicity. The change can be seen in line 11.**

- RQ in abstract: what are the associated factors with gender incongruence and its levels of evidence in the scientific literature? It’s not clear to me what ‘its’ refers to and ‘levels of evidence’ seems to need definition. See my below comment in Methods for suggested rephrasing.

**Answer: We appreciate this suggestion. We have decided to take it as proposed by Reviewer #1 in the Methods. The change can be seen in lines 14-15.**

- You have made no mention of planned analysis method. I think some indication should be given here.

**Answer: We appreciate the suggestion. We have carefully reviewed more than 20 scoping review protocols published in PLOS ONE and none of them detail the method of analysis in the abstract (1-20, see References at the bottom). Reviewing the official JBI guidelines on Scoping Review protocols (https://jbi.global/scoping-review-network/resources), it does not appear as necessary to make reference to the method of analysis. However, in order to increase clarity, we have decided to add information about how the extracted data will be used and how they will be presented, in lines 21-24.**

- Overall I don’t think the abstract goes far enough to describe methods or even make a case for how it could be useful. It is just too vaguely stated at present.

**Answer: As in the response to the previous suggestion, none of the Scoping Review protocols reviewed provide an abstract with more detail regarding the results and their usefulness. The JBI guidelines do not state that abstracts should have more detail. However, we believe that the clarifications we have added about how we will use the extracted data and its presentation make our abstract more complete. Finally, with regard to arguing for the usefulness of this review, we have found it appropriate to make it even more explicit that evidence-based knowledge about the factors of gender incongruence are of great use to the clinician, considering that such factors can influence gender identity and decision making.**

Introduction

- p8, para 3: ‘In the review prior to the development of this protocol’ – not clear what this refers to. Is this your own lit search in planning the present study? In which case, make that more clear.

**ANSWER: We appreciate the suggestion. We have decided to reword the above-mentioned sentence more clearly. The change can be seen in line 61.**

- Overall very clear and well written.

**ANSWER: Thank you!**

Method

- p9, para 2 – couple of grammatical tweaks. Point (III) should read ‘chart’ not ‘charting’, and the last clause, ‘as will be depicted as follows’ doesn’t make sense.

**ANSWER: We appreciate the suggestion. We have decided to reword the above-mentioned sentence more clearly. The change can be seen in lines 99-100.**

- I RQ – re-phrase first sentence – ‘The question that arises from the above is the next:…’.

**ANSWER: We have decided to rephrase the above-mentioned sentence more clearly. The change can be seen in lines 103-104.**

- I RQ – RQ itself also needs to be rephrased. Currently: What are the factors associated with gender incongruence and its levels of evidence in the scientific literature? Suggest changing to ‘What are the factors associated with gender incongruence and what level of evidence is there for each factor in the scientific literature?

**ANSWER: Excellent observation. We have decided to change the RQ using the same words that reviewer N°1 suggested. It can be seen in lines 104-105.**

- Pubmed and Scopus, although large, are not likely to generate all the relevant literature. What about PsycINFO and CINAHL? You may be interested in two of my papers published in PLOS Global Public Health, which is currently too new to be indexed. (Thompson et al ‘A PRISMA systematic review of adolescent gender dysphoria literature’ – parts 1 and 2 published, part 3 under review.) Please don’t take this to mean I expect you to cite these – this is not the point. I just want to say there may be relevant papers outwith Pubmed and Scopus so I would seriously consider casting a wider net.

**ANSWER: We think this suggestion is very appropriate. We have decided to include both databases proposed by the reviewer. It is important to note that this change will imply a change in the flow chart (Fig1). In addition, we have reviewed the referred articles, and at least one of them has seemed to us totally relevant to our research, so we have decided to include it in the literature reviews related to gender incongruence (line 64).**

- ‘In addition, the cited sources will be reviewed to capture gray literature and find keywords that guide the final literature search.’ I am not sure this would be sufficient. For grey literature, authors should consider also using databases especially designed around this literature, e.g., OpenDOAR, PsycEXTRA.

**ANSWER: Considering the large number of articles that will result from the search of the 4 databases included, we have decided not to include gray literature on this occasion.**

- p9 – explicate PCC in reference to mnemonic criteria

**ANSWER: mnemonic explained.**

- search strategy (appendix 1) – should you include the alternative spelling ‘aetiology’ as well?

**ANSWER: Excellent idea. We have incorporated the word "aetiology" in the search strategy.**

- Search strategy (appendix 1) – I appreciate this will be a preliminary search, but I suggest including terms such as ‘gender nonconforming’ ‘gender reassignment’ ‘sex reassignment’ and ‘transsexual’.

**ANSWER: We appreciate this observation. It seems appropriate to include "transsexual", "sex reassignment" and "gender reassignment" in the search strategy. Regarding the term "gender nonconforming", we actually considered including it. However, in reviewing some articles it has seemed to us that this is a difficult gender identity to assess, unlike gender incongruence. Indeed, within the concept of "gender nonconforming" there is room not only for those who define themselves as transgender, but also for people who are agender, gender non-binary, etc. This openness to such different situations seems to us to obscure the focus of this review, although they could be addressed in future research.**

- ‘of examining the reference lists before carrying out an intensive search in both repositories’ – suggest change to ‘hand search’ and it’s not clear what is meant by an intensive search of both repositories. Does it mean your search terms may be further augmented?

**ANSWER: We appreciate this suggestion. It seems to us that the third phase of the study selection was worded in an ambiguous way, so we have rewritten it. Along with this, we have added more detail in the first and second phases as well. These changes can be seen in lines 145-150.**

- Resolving disagreement by discussion – is there a third person who could be brought in where it’s difficult to reach agreement?

**ANSWER: Excellent point. It has been included on line 157.**

- Fig 1 image quality is poor.

**ANSWER: Image quality has been improved. Also, it has been modified because of the inclusion of two additional databases.**

- p11 – levels of evidence. This hierarchy seems reasonable, but I wonder if you will be using a systematic quality assessment tool as well. That is, you could have a paper that reports an RCT (so rated 2 on level of evidence) but the quality of the study / paper is not very good. How will this be dealt with? There are good tools for assessing the quality of studies of diverse designs, such as the Crowe Critical Appraisal Tool (CCAT) or the Quality Assessment Tool for Studies of Diverse Designs (QATSDD).

**ANSWER: Excellent suggestion. The truth is that we would love to be able to evaluate the quality of the chosen studies. However, I am afraid that on this occasion it will not be possible for us. First of all, because the evaluation of the quality of the articles is not allowed within the scoping review format (more information in https://jbi-global-wiki.refined.site/space/MANUAL/4687794/11.1.1+Why+a+scoping+review%3F). The objective of the scoping review is more humble: to map what is available, describe the gaps in the literature and prepare future systematic reviews where the quality of the studies is evaluated. Secondly, because this would involve work that exceeds the current possibilities of the research team. The assessment of the quality of the articles could be done in a future systematic review.**

Results – I appreciate the need to be vague without knowing the nature of the findings. But I think you could say that the study characteristics will be given in a table (you could even provide headings), and that you will endeavour to find a way to present the factors identified cross-referenced with their level of evidence AND an assessment of the quality of the literature (see my previous comment).

**ANSWER: This comment seems appropriate. Although the Scoping Review protocols normally do not include this section, we thought it would be interesting to mention some aspects suggested in this commentary, such as the way in which the results will be presented and even the attempt we will make to cross-reference the types of factors with the levels of evidence. As a consequence, we have restructured the "results" section. Regarding the evaluation of the quality of the literature, as we explained in the previous response, it will not be possible to do so on this occasion.**

Contributions statement – I’m not sure ‘redacted’ is the term you want here. Drafted and edited?

**ANSWER: Based on this comment and other indications given by the guest editor, we have restructured the contribution statement and the problem noted in this comment is no longer present.**

Reviewer #2: General comments:

- Figure 1 is quite blurry at the current uploaded resolution

**ANSWER: Image quality has been improved.**

- etiology is almost certainly multifactorial, though I agree a scoping review could summarize potential contributors

**ANSWER: Ok.**

Research question:

- Regarding this sentence, "Finally, it is hoped that the results of this research (4) may be useful for therapists...," WPATH SOC version 8 has removed the requirement that therapists are the only providers who have to evaluate and provide treatment recommendations for gender incongruence. It may be more accurate to change "therapists" to "clinicians evaluating gender incongruence"

**ANSWER: We appreciate this observation. We have changed the manuscript as suggested, on line 27.**

Methods:

- With regards to the data extraction from included studies, it's unclear to me how the "methodological design" will be different from the "levels of evidence" since you are essentially substituting the method design for levels of evidence anyways.

**ANSWER: Thank you for your comment. Indeed, "methodological design" and "levels of evidence" seem to be similar. However, they are concepts that refer to different things: the methodological design describes the way a research is conducted, while the level of evidence refers to the quality of the content obtained from the research. This difference could be compared to saying that a day is "sunny" or that it is "hot": both are valid ways to describe a day, but one puts more emphasis on the intensity of the sun and the other on the subjective sensation of that intensity. In summary: it is true that the level of evidence is deduced from the methodological design, but this does not mean that they are identical in content.**

**In our case, we have found the concept of "level of evidence" more appropriate for three reasons: (1) We wish to place more emphasis on the quality of the evidence than on the type of methodology. (2) Each level of evidence groups together various types of methodological design with aspects in common, and is therefore a somewhat more summarized classification system than the list of methodological designs. (3) The levels of evidence imply a comparison between products of methodological designs, which allows us to establish a "ranking" of studies according to their quality.**

Discussion:

- Regarding this sentence in your discussion: "One of its key points states: "There is lack of consensus and open discussion about the nature of gender dysphoria and therefore about the appropriate clinical response" [36]." I disagree that there is lack of consensus around the appropriate clinical response - the appropriate clinical response is to support trans/gender diverse individuals and there is evidence base for gender-affirming treatments in reducing mental health symptoms, improving quality of life, etc.

**ANSWER: We appreciate the comment. We have decided to remove the entire paragraph because (1) we understand that the literal quote from Dr. Hillary Cass may cause misunderstandings and (2) after discussion it seemed to us that it is not necessary to go into this issue to properly meet the objectives of our protocol.**

- With regards to this sentence in your discussion: "Understanding the etiology of gender dysphoria, would help clinicians decide which type of intervention will be helpful in each case, and might also help to prevent regret and detransition, which appears to be becoming increasingly common [37, 38]," your included references do not state that regret and/or detransition are becoming more common and they do not even speak to the incidence/prevalence. Rather those two cited studies seek to understand the phenomena of detransition. The last part of this sentence should be changed - as written, it is not supported by references and seems provocative.

**ANSWER: It seems pertinent to us to remove the last part of the sentence, as suggested.**

- I also wonder if you could speak to how interventions might differ due to the potential etiology of gender incongruence?

**ANSWER: We appreciate this observation. We have decided to re-elaborate the last section of the discussion to make it more explicit how a better understanding of the etiology of gender incongruence might, in each case, help in choosing the best first-line intervention.**

7. PLOS authors have the option to publish the peer review history of their article (what does this mean?). If published, this will include your full peer review and any attached files.

If you choose “no”, your identity will remain anonymous but your review may still be made public.

Do you want your identity to be public for this peer review? For information about this choice, including consent withdrawal, please see our Privacy Policy.

Reviewer #1: Yes: Lucy Thompson

Reviewer #2: No

**ANSWER: We appreciate the time and dedication of each of the reviewers. We hope that our responses are clear enough to satisfy your concerns. We are confident that, thanks to your suggestions, we will have the opportunity to submit a manuscript of a much higher quality than the one we originally submitted.**

**References**

1. Shah YB, Shaver AL, Kelly WK, Lu-Yao G. A scoping review protocol to elucidate outcomes following abiraterone versus enzalutamide for prostate cancer. PLOS ONE. 2022 Aug 29;17(8):e0273826.
2. Parker M, Fang X, Self-Brown SR, Rahimi A. Establishing how social capital is studied in relation to cardiovascular disease and identifying gaps for future research—A scoping review protocol. PLOS ONE. 2021 Apr 8;16(4):e0249751.
3. Amri M, Ali S, Jessiman-Perreault G, Barrett K, Bump JB. Evaluating healthy cities: A scoping review protocol. PLOS ONE. 2022 Oct 20;17(10):e0276179.
4. Krzeczkowski JE, Wade TJ, Andrade BF, Browne D, Yalcinoz-Ucan B, Riazi NA, et al. Examining the mental health of siblings of children with a mental disorder: A scoping review protocol. PLOS ONE. 2022 Sep 15;17(9):e0274135.
5. Kavanagh BE, Beks H, Versace VL, Quirk SE, Williams LJ. Exploring the barriers and facilitators to accessing and utilising mental health services in regional, rural, and remote Australia: A scoping review protocol. PLOS ONE. 2022 Dec 9;17(12):e0278606.
6. Spreuwel PCJM van, Munk-Overkamp MLC, Smit LC, Ziesemer KA, Loveren C van, Heijden GJMG van der, et al. Exploring the development, evaluation and implementation of complex health interventions to prevent early childhood caries in preschool children: A scoping review protocol. PLOS ONE. 2022 Oct 10;17(10):e0275501.
7. Lubeya MK, Mwanahamuntu M, Chibwesha C, Mukosha M, Wamunyima MM, Kawonga M. Implementation strategies to increase human papillomavirus vaccination uptake for adolescent girls in sub-Saharan Africa: A scoping review protocol. PLOS ONE. 2022 Aug 25;17(8):e0267617.
8. Sia D, Nguemeleu ET, Beogo I, Séguin C, Roch G, Cleveland J, et al. Interventions facilitating access to perinatal care for migrant women without medical insurance: A scoping review protocol. PLOS ONE. 2022 Mar 14;17(3):e0265232.
9. Nwagbara UI, Osuala EC, Chireshe R, Babatunde GB, Okeke NO, Opara N, et al. Mapping evidence on factors contributing to maternal and child mortality in sub-Saharan Africa: A scoping review protocol. PLOS ONE. 2022 Aug 10;17(8):e0272335.
10. Lamont-Mills A, Bayliss LT, Christensen SA. Online suicidal thoughts and/or behaviours talk: A scoping review protocol. PLOS ONE. 2022 Oct 27;17(10):e0276776.
11. Nunes J de O, Domingues RAS, Junior RMPCB, Alves BMCS, Carvalho IPSF de, Tavares NUL. Policy and strategies addressing prevention and control of antimicrobial resistance in Brazil: A scoping review protocol. PLOS ONE. 2022 Jan 28;17(1):e0263305.
12. Widyaningsih V, Febrinasari RP, Sari V, Augustania C, Verlita B, Wahyuni C, et al. Potential and challenges for an integrated management of tuberculosis, diabetes mellitus, and hypertension: A scoping review protocol. PLOS ONE. 2022 Jul 12;17(7):e0271323.
13. Iwu-Jaja CJ, Jaca A, Jaja IF, Jordan P, Bhengu P, Iwu CD, et al. Preventing and managing antimicrobial resistance in the African region: A scoping review protocol. PLOS ONE. 2021 Jul 14;16(7):e0254737.
14. Hirani S, Ojukwu E, Bandara NA. Prosocial behavior and youth mental health outcomes: A scoping review protocol. PLOS ONE. 2022 Jun 24;17(6):e0270089.
15. Machado LR, Silva CFR da, Hadders-Algra M, Tudella E. Psychometric properties of the Infant Motor Profile (IMP): A scoping review protocol. PLOS ONE. 2022 Nov 16;17(11):e0277755.
16. Karteczka-Świętek K, Opozda-Suder S, Strojny A. Psychosocial functioning of adolescents with ADHD in the family, school and peer group: A scoping review protocol. PLOS ONE. 2022 Jun 17;17(6):e0269495.
17. Martarelli R, Casanova G, Lamura G. The impact of long-term care needs on the socio-economic deprivation of older people and their families: A scoping review protocol. PLOS ONE. 2022 Aug 31;17(8):e0273814.
18. Senapati RE, Parida J, Badamali J, Jena S, Patsani P, Panda A, et al. The patterns, trends and major risk factors of suicide among Indian adolescents—A scoping review protocol. PLOS ONE. 2022 Nov 17;17(11):e0277422.
19. Steensgaard R, Hundborg MO, Pallesen H, Aadal L. What is a meaningful life for persons with acquired neurological impairments? A scoping review protocol. PLOS ONE. 2022 Jun 16;17(6):e0269125.
20. Philippe K, Perrotta C, O’Donnell A, McAuliffe FM, Phillips CM. Why do preconception and pregnancy lifestyle interventions demonstrate limited success in preventing overweight and obesity in children? A scoping review protocol. PLOS ONE. 2022 Nov 3;17(11):e0276491.
